# The functional activity of donor kidneys is negatively regulated by microribonucleic acid-451 in different perfusion methods to inhibit adenosine triphosphate metabolism and the proliferation of HK2 cells

**DOI:** 10.1080/21655979.2022.2068739

**Published:** 2022-05-21

**Authors:** Xu-Hui Zhu, Long-Xi Han, Rong-Jie Zhang, Peng Zhang, Fu-Gang Chen, Jia Yu, Heng Luo, Xiu-Wu Han

**Affiliations:** a Department of Urology, Beijing Chao-yang Hospital, Capital Medical University, Beijing, PR China; b Department of General Surgery, Guizhou Provincial Staff Hospital, Guiyang PR China; c State Key Laboratory of Functions and Applications of Medicinal Plants, Guizhou Medical University, Guiyang PR China; d The Key Laboratory of Chemistry for Natural Products, Guizhou Province and Chinese Academy of Science, Guiyang PR China

**Keywords:** Donor kidney, RNA-sequencing, miRNA marker, different perfusion preservation methods

## Abstract

This study explored the regulation of different perfusion methods on ischemia-reperfusion injury in donor kidneys. In this study, renal cortical/medullary tissue specimens were collected from porcine kidneys donors using different perfusion methods at various time points. Hematoxylin and eosin (H&E) staining was used to test the histological differences. Differentially expressed micro-ribonucleic acids (miRNAs) were identified by miRNA transcriptome sequencing. Reverse transcription-polymerase chain reaction (RT-PCR) tests were used to verify the changes in miRNAs in the kidney tissue taken from different perfusion groups. The related signaling pathways and the changes in the cell functions of different perfusion groups were analyzed by Kyoto Encyclopedia of Genes and Genomes (KEGG) /Gene Ontology (GO) bioinformatics analyses. The effects of miRNA overexpression on the metabolism and proliferation of HK2 cells were detected by ATP kit and MTT assay. The H&E staining results showed that there were essentially no differences in the tissue samples among different perfusion groups at and before 12 h compared with a control group. The quantitative PCR results revealed that there was essentially no change in the expression of ssc-miR-451, ssc-miR-1285, and ssc-miR-486 in the cis infusion or joint infusion kidney groups, and their expression was significantly down-regulated over time in the trans-infusion kidney group. The bioinformatics analysis showed that the cellular component, molecular function, and biological processes of the kidney tissue, which had been perfused using three methods, had been consistently affected. The most significant changes after perfusion occurred in the intracellular metabolism signaling pathways. Furthermore, the energy metabolism and proliferation of the HK2 cells were significantly inhibited after the overexpression of miR-451. Specific miRNA markers, such as miR-451, may play a negative regulatory role in cell metabolism following the perfusion of kidney transplants using different methods.

## Graphical abstract

**Figure:**
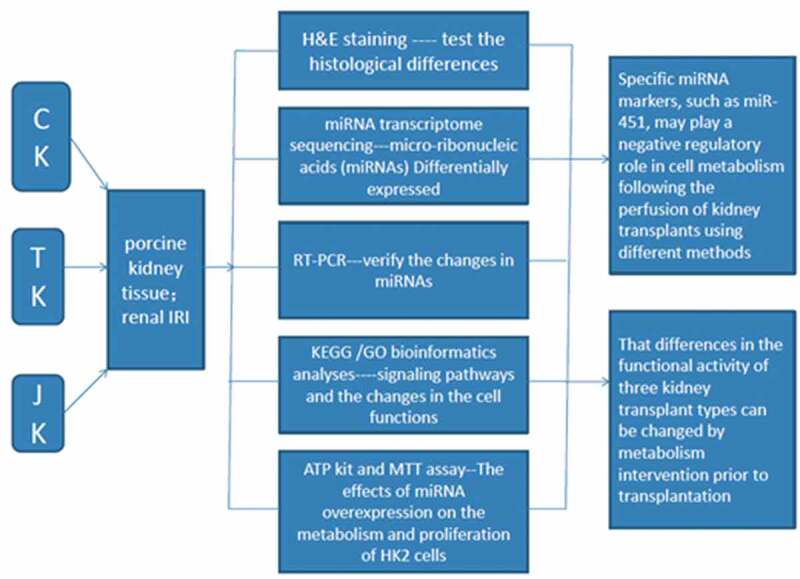

## Highlights

- This study identified miRNAs as molecular markers with specific expression and a conservative function during perfusion via the high-throughput sequencing of miRNAs in porcine kidney tissue, which was subjected to different perfusion methods to determine the regulation of these methods on renal IRI.
- The energy metabolism and proliferation of the HK2 cells were significantly inhibited after the overexpression of miR-451.
- Specific miRNA markers, such as miR-451, may play a negative regulatory role in cell metabolism following the perfusion of kidney transplants using different methods.
- The results suggested that the essentially equivalent changes in early tissue samples and at molecular levels, as well as similar changes in cell functioning and signaling pathways, may serve as an internal mechanism for the mutual replacement of three different perfusion methods, and that differences in the functional activity of three kidney transplant types can be changed by metabolism intervention prior to transplantation.

## 1. Introduction

In recent years, with the preservation and rational use of transplanted organs and the application of potent post-transplant immunosuppressants, the short-term and mid-term survival rates and quality of life of kidney transplant recipients have been significantly improved; however, assurance of their long-term survival has not followed the same trend. Ischemia-reperfusion injury (IRI), organ-rejection reactions, as well as immune tolerance are key factors affecting long-term graft survival [1].

Renal IRI is a common complication following kidney transplantation that mainly occurs in the microvascular network and is a primary cause of acute renal failure. In kidney transplantation cases, after the in vitro-preserved donor kidney is transplanted to the recipient and blood supply is restored, excessive free radicals will attack the cells in selected tissue to which the blood supply is restored, causing injury. The effective protection of the donor kidneys during transplantation is paramount for reducing renal IRI.

In the current kidney transplant process, the hypothermic machine perfusion–cis-infusion kidney (CK–the hypothermic machine perfused flow is from artery to vein of kidney) method is generally considered the most effective for protecting the activity of the donor kidneys in vitro [2]. The research team that conducted the present study has long been engaged in the exploration of kidney transplantation. Studies in this area have demonstrated that in addition to CK, the hypothermic machine perfusion–trans-infusion kidney (TK) (TK – the hypothermic machine perfused flow is from vein to artery of kidney, where the perfused flow is the opposite to that of CK) and the joint-infusion kidney (JK – combined with CK and TK) methods can also effectively protect the functioning of donor kidneys, and their protective effect is superior to that of using CK only. However, the molecular mechanisms that are involved in these methods remain unclear.

The pre-transplant changes in the molecular biomarker levels of donor kidneys are critical to the functional evaluation of transplanted kidneys and for ensuring the graft’s survival. These changes also have significant reference and popularization value due to their quick and easy detection and strong reliability. Related articles reported that the functional evaluation factors of renal IRI during transplantation include the following: (1) a renal protective factor, i.e., heme oxygenase-1 (HO-1) and (2) a kidney injury molecule, i.e., (KIM-1) [3–6]. As potential noninvasive diagnostic biomarkers, the identification of some micro-ribonucleic acids (miRNAs) presents new approaches for diagnosing and identifying kidney transplantation failures. Ardalan et al. investigated the plasma specimens of 53 kidney transplant recipients, including those with long-term stable graft function (N = 27), recipients with biopsy results that showed interstitial fibrosis and renal tubular atrophy (N = 26), and a healthy control group (N = 15); they found that the levels of miR-21, miR-142-3p, and miR-155 were associated with renal insufficiency and could be used for transplantation monitoring [7]. Wei et al. identified nine miRNAs (miR-142-5p, miR-142-3p, miR-223, miR-211, miR-486, miR-155, miR-10b, miR-30a, and let-7c) related to human kidney transplantation status in 104 kidney transplant recipients, suggesting that the differential expression of MiRNAs in human peripheral blood mononuclear cells could help predict the functioning of transplanted kidneys [8]. Currently, most existing studies on the molecular biomarkers of kidney donors have focused on the postoperative IRI and monitoring processes, and a few have focused on the target biomarkers for functional evaluation during the donor kidney perfusion preservation period.

To explore the regulation of different perfusion methods on IRI in donor kidney cases, in the current preliminary study, this research group used Guizhou Jianhe White Xiang strain II pigs (jointly bred and raised by the China Agricultural University and Guizhou University) as experimental animals to investigate the effects of different perfusion methods on the activity of kidney tissue. These pigs have very similar characteristics to humans concerning physiological function, e.g., the shape and size of organs, and are characterized by strong adaptability and disease resistance. They are also cost-effective to breed and, as such, are ideal organ donors for human xenotransplantation.

Based on the results, the TK and JK methods showed good protective effects on the donor kidneys. However, the regulation mechanism and the in vivo effectiveness of the three perfusion protection methods on the IRI of the donor kidneys remained unclear. This study aims to identify miRNAs as molecular markers with specific expression and a conservative function during perfusion via the high-throughput sequencing of miRNAs in porcine kidney tissue, which was subjected to different perfusion methods to determine the regulation of these methods on renal IRI. And to know that differences in the functional activity of three kidney transplant types can be changed by metabolism intervention prior to transplantation.

## 2. Materials and methods

### 2.1 Renal ischemia-perfusion of kidney tissue

Guizhou Jianhe White Xiang strain II pigs were used as experimental animals and were subjected to the following different perfusion operations after grouping. 1) A no perfusion kidney (NK) group (negative control); 2) a cis-infusion kidney (CK) group (positive control); 3) an experimental trans-infusion kidney (TK) group; and 4) an experimental joint-infusion kidney (JK) group. Each group included 3 pigs, and total number of pigs was 12. Kidney tissue samples were collected at 0, 1, 6, 12, 18, and 24 h after perfusion and cryopreserved in liquid nitrogen for later use. The perfusion experiment was jointly performed by Beijing Chao-Yang Hospital, Capital Medical University, Guizhou Provincial Staff Hospital, and the Key Laboratory of Chemistry for Natural Products of Guizhou Province and Chinese Academy of Sciences. Animal specimens were kept at the Key Laboratory of Chemistry for Natural Products of Guizhou Province and Chinese Academy of Sciences. This study was approved by the Ethics Committee of Beijing Chao-Yang Hospital, Capital Medical University.

### 2.2 Hematoxylin and eosin staining

Tissue blocks were stored in liquid nitrogen, fixed with 4% paraformaldehyde, embedded in conventional paraffin, and sectioned into a thickness of 4–5 µm (using a Thermo Finesse E+ manual slicer). The sections were prepared as follows: (1) deparaffinated with xylene: xylene I for 15 min, xylene II for 15 min, xylene:absolute alcohol = 1:1 for 2 min; (2) reconstituted with gradient alcohol: 100% ethanol I for 5 min, 100% ethanol II for 5 min, 80% ethanol for 5 min, and distilled water for 5 min; (3) stained with a hematoxylin solution for 5 min and washed with water for 10 min, or flushed with running water for 5 min; (4) differentiated with acid and water: 1% hydrochloric acid alcohol for 30s, washed with water for 30s, and washed with distilled water for 5 s; (5) stained with a 0.5% eosin solution for 1–3 min and washed with distilled water for 30s; (6) dehydrated with gradient alcohol: 80% ethanol for 30s, 95% ethanol I for 1 min, 95% ethanol II for 1 min, absolute alcohol I for 3 min, and absolute alcohol II for 3 min; (7) transparentized with xylene: xylene I for 3 min and xylene II for 3 min; (8) sealed with a neutral resin; (9) observed and photographed with an Olympus BX41 microscope.

### 2.3 The miRNA sequencing

Approximately 2–4 g of kidney tissue that had been sampled from each group and cryopreserved in liquid nitrogen at 0 and 12 h was placed in Eppendorf tubes and clearly marked, wrapped with sealing film, and sent with dry ice to Guangzhou RiboBio Co., Ltd. for miRNA sequencing. The sequencing samples of each group were recorded as follows: 1) the NK group: NK1-0, NK2-0, NK1-12, NK2-12; the CK group: CK1-0, CK2-0, CK1-12, CK2-12; the TK group: TK1-0, TK2-0, TK1-12, TK2-12; the JK group: JK1-0, JK2-0, JK1-12, JK2-12. The total RNA fragment of each sample was extracted and successively connected with the 3’-end and 5’-linker, then reverse-transcribed into complementary deoxyribonucleic acid (cDNA) and amplified by a polymerase chain reaction (PCR). The target fragment library was cut and collected and sequenced after passing a quality inspection. Sequencing was carried out using the SE50 on the Illumina Hiseq 2500 sequencing platform. Raw reads obtained from the sequencing were filtered as follows: 1) adapters at both ends of the reads; 2) reads showing a length of <17 nt; the low-quality reads were removed to complete their preliminary filtration to get clean reads. Clean reads within the sequencing reads were compared with reference genomes using the BWA software to obtain the genome-wide reads distribution map and annotated by non-coding RNA classification [9–11]. The identified miRNAs were subject to the calculation of their expression level, an expression clustering analysis, and differential expression analysis among the samples. Significantly differentiated miRNAs were further predicted for their target genes and Gene Ontology (GO) and Kyoto Encyclopedia of Genes and Genomes (KEGG) biological pathway enrichment analyses were carried out for the target genes.

### 2.4 Venn diagram

Differentially expressed genes in the 0 and 12 h sequencing results of the perfusion treatment groups (i.e., the CK, TK, and JK groups) were cross-compared to analyze the association between the differentially expressed gene sets among the three groups. The Venny tools online software provided support for this process (https://bioinfogp.cnb.csic.es/tools/venny/index.html).

### 2.5 Real-time quantitative PCR

The kidney tissue of each group that had been cryopreserved in liquid nitrogen was ground on ice, and the total RNA was extracted using a TRIzol® reagent (Invitrogen, USA). An equivalent amount of RNA was subjected to reverse transcription (RT) into cDNA using a HiFiScript kit (cDNA synthesis kit, CWBIO, China) and amplified by PCR (CWBIO, China). Real-time quantitative PCR (qPCR) was carried out on an RT-PCR system called StepOnePlus™ (Thermo Fisher Scientific, USA) using an UltraSYBR kit (CWBIO, China). The assay was conducted in 15 µL of the final reaction system according to the following scheme: initial denaturation at 95°C for 10 min, 95°C for 15s, 60°C for 1 min, and 95°C for 15s, for a total of40 cycles; at the melting curve phase at 60°C for 1 min, 95°C for 15s, and 60°C for 15s. Taking U6 as the control, the primer sequence is shown in Table 1.

**Table 1.** qRT-PCR primer sequence list

A. qRT-PCR primer sequence in pig		
Gene	Sequence(5’-3’)	
ssc-miR-451 RT primer	GTCGTATCCAGTGCAGGGTCCGAGGTATTCGCACTGGATACGACAACTCA	
ssc-miR-451 FW	GCCCGCaaaccgTTaccaTTacT	
ssc-miR-451 RV	ATCCAGTGCAGGGTCCGAGG	
ssc-miR-486 RT primer	GTCGTATCCAGTGCAGGGTCCGAGGTATTCGCACTGGATACGACCTCGGG	
ssc-miR-486 FW	TccTgTacTgagcTgccccgag	
ssc-miR-486 RV	ATCCAGTGCAGGGTCCGAGG	
ssc-miR-1285 RT primer	GTCGTATCCAGTGCAGGGTCCGAGGTATTCGCACTGGATACGACACGGGG	
ssc-miR-1285 FW	CTgggcaacaTagcgagaCCC	
ssc-miR-1285 RV	ATCCAGTGCAGGGTCCGAGG	
ssc-U6 FW	CCCTTCGGGGACATCCGATA	
ssc-U6 RV	TTTGTGCGTGTCATCCTTGC	
B. qRT-PCR primer sequence in human		
Gene		Sequence(5’-3’)
Hsa-miR-451-5p RT primer		GTCGTATCCAGTGCAGGGTCCGAGGTATTCGCACTGGATACGACAACTCA
Hsa-miR-451-5p FW		CCCGAAACCGTTACCATTACTG
Hsa-miR-451-5p RV		ATCCAGTGCAGGGTCCGAGG
Hsa-miR-486-5p RT primer		GTCGTATCCAGTGCAGGGTCCGAGGTATTCGCACTGGATACGACCTCGCG
Hsa-miR-486-5p FW		TCCTGTACTGAGCTGCCCCGAG
Hsa-miR-486-5p RV		ATCCAGTGCAGGGTCCGAGG
Hsa-miR-1285-3p RT primer		GTCGTATCCAGTGCAGGGTCCGAGGTATTCGCACTGGATACGAC
Hsa-miR-1285-3p FW		TCTGGGCAACAAAGTGAGACCT
Hsa-miR-1285-3p RV		ATCCAGTGCAGGGTCCGAGG

### 2.6. Cell culture

Human renal tubular epithelial cells (HK2) were purchased from Chinese Academy of Sciences, Shanghai Institute of Biochemistry and Cell Biology, and the cells were cultured in Dulbecco’s Modified Eagle Medium (DMEM) = (Cellmax). All media contained 10% fetal bovine serum (FBS), 100 U/mL penicillin and streptomycin (Invitrogen). The cells were confirmed that they did not contain mycoplasma and were incubated with 5% CO2.

### 2.7 Adenosine triphosphate activity assays

The culture cells were collected after transfection for 24 h. The activities of adenosine triphosphate (ATP) were assayed using ATP kits (Nanjing Jiancheng Bioengineering, China) according to the manufacturer’s instructions.

### 2.8 Methyl thiazolyltetrazolium (MTT) assay

The methyl thiazolyltetrazolium (MTT) assay was used to evaluate the HK2 cell proliferation following miRNA overexpression. The cell culture was prepared according to the method described above. Then, the HK2 cells were seeded in 24-well plates at a density of 1 × 10^4^–5 × 10^4^ cells/well and incubated for 24 h. The cells were exposed for 48 h after the transfection of miRNA mimics and evaluated under inverted microscopic equipment (Leica, Japan). The MTT (Solarbio, Beijing) solution (5 mg/mL, dark) was added to each well and incubated at 37°C for 4 h. The supernatant was removed and 500 μL dimethylsulfoxide (Solarbio, Beijing) was added. The plates were gently shaken to dissolve blue formazan crystals and the absorbance was tested at 490 nm using a microplate reader (Gene, Hong Kong, China).

### 2.9 Statistical analysis

All data were processed by GraphPad Prism 6 software. A two-way analysis of variance and a Student’s T-test, respectively, were performed; P < 0.05 was considered to have statistical significance, and P < 0.01 was considered to indicate an extremely significant difference.

## 3. Results

This study aims to identify miRNAs as molecular markers with specific expression and a conservative function during perfusion via the high-throughput sequencing of miRNAs in porcine kidney tissue, which was subjected to different perfusion methods to determine the regulation of these methods on renal IRI.

The results of this study suggest that the essentially equivalent changes in early tissue samples and at molecular levels, as well as similar changes in cell functioning and signaling pathways, may serve as an internal mechanism for the mutual replacement of three different perfusion methods, Specific miRNA markers, such as miR-451, may play a negative regulatory role in cell metabolism following the perfusion of kidney transplants using different methods. And that differences in the functional activity of three kidney transplant types can be changed by metabolism intervention prior to transplantation.

### 3.1 The effects of different perfusion methods on donor kidney tissue morphology

**Figure 1.**
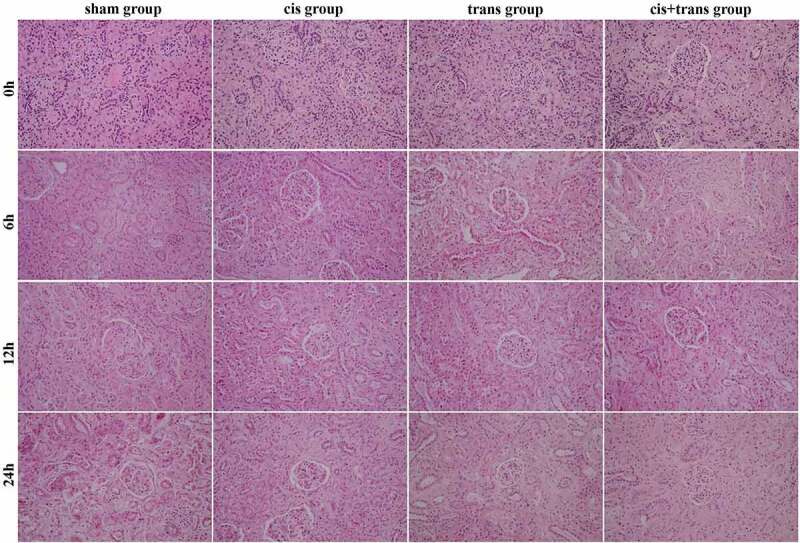
The H&E staining results. Eyepiece x10 + objective x20. The experiment was repeated three times independently.

To explore the effects of different perfusion methods on kidney tissue injury, hematoxylin and eosin (H&E) staining was utilized to detect the renal cortex/medulla tissue layer of the pigs in different perfusion groups. The H&E pathological score results indicated moderate-to-severe renal tubular degeneration and atrophy in all groups, and mild inflammatory cell infiltration was observed in a few tissue samples. Mild-to-moderate interstitial fibrous hyperplasia was also observed in individual tissue samples. At 0 h, there was no obvious difference in the tissue structure among the four groups. At 6 h, the perfusion groups had increased tissue space compared with the control group. At 12 h, there was essentially no difference among the groups. At 24 h, the perfusion groups showed few changes and a complete tissue structure, while the control group had more serious renal tubular atrophy (Figure 1). The above results suggested that the histopathological findings of the transplanted kidneys in different perfusion methods were consistent; there were small pathological differences among the different perfusion groups.

### 3.2 The MiRNA-sequencing and qRT-PCR verification of donor kidney tissue using different perfusion methods

**Figure 2.**
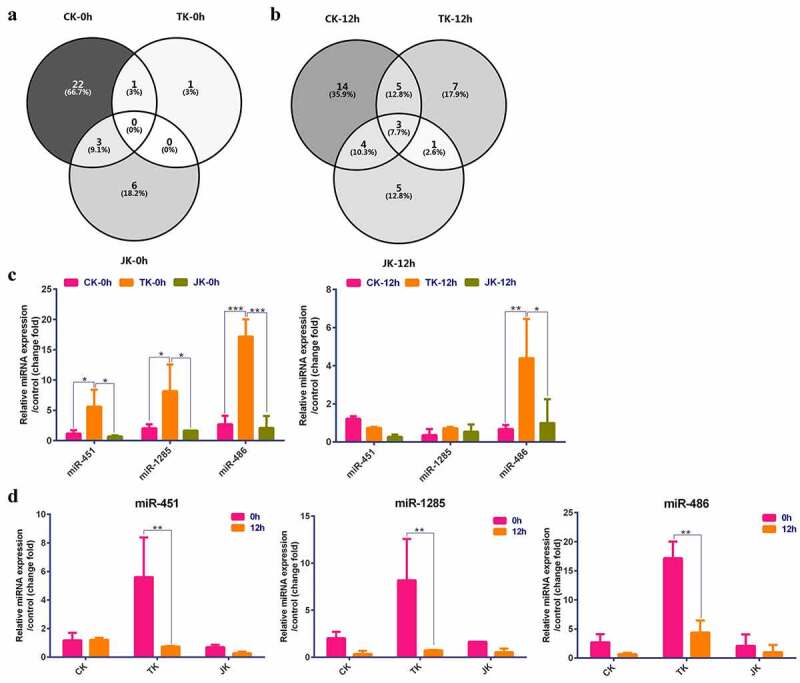
The Venn diagrams and qPCR results. (a–b) Venn diagrams of the CK, TK, and JK groups at 0 and 12 h. C. The qPCR verification results of miR-451, miR-1285, and miR-486 in kidney tissue samples taken from different perfusion groups at 0 and 12 h. D. The qPCR results of miR-451, miR-1285, and miR-486 in kidney tissue samples taken from different perfusion groups at 0 and 12 h. The experiment was repeated three times independently. Compared with the negative control group, * P < 0.05, **P < 0.01.

To continue studying changes at the molecular level of the kidney tissue when using different perfusion methods, miRNA high-throughput sequencing was performed on the tissue samples of all the groups collected at 0 and 12 h. The miRNA sequencing results (Figure 2a) revealed that, compared with the NK group, there was no overlap of differential miRNAs between the three groups when using the different perfusion methods at 0 h. Among the differential miRNAs, a differential expression of ssc-miR-432-5p was found in the CK and TK groups. Compared with the CK group, there were fewer differentially expressed genes in the TK group, and ssc-miR-451 was a differentially expressed gene in this group. The differential expression of ssc-miR-381-3p, ssc-miR-216, and ssc-miR-33b-3p was found in both the CK and JK groups. Compared with the CK group, differentially expressed genes were significantly decreased, and six specifically expressed genes were additionally identified in the JK group.

At 12 h after perfusion, three genes, i.e., ssc-miR-451, ssc-miR-486, and ssc-miR-1285, were differentially expressed in the three groups using different perfusion methods (Figure 2b). In addition, ssc-miR-451, ssc-miR-450a, ssc-miR-486, ssc-miR-132, ssc-miR-212, ssc-miR-155-5p, ssc-miR-146b, and ssc-miR-1285 were differentially expressed in both the CK and TK groups. The TK group had more differentially expressed genes than at 0 h and had an additional seven differentially expressed genes compared with the CK group. The genes ssc-miR-451, ss-miR-486, ssc-miR-7140-3p, ssc-miR-19b, ssc-miR-20a-5p, ssc-miR-935, and ssc-miR-1285 were differentially expressed in both the CK and JK groups. The JK group had more differentially expressed genes than at 0 h and had an additional six differentially expressed genes compared with the CK group.

In this study, three common differential genes in the demarcated groups, i.e., ssc-miR-451, ssc-miR-486, and ssc-miR-1285, were verified by qPCR assays on the tissue samples at 0 and 12 h after perfusion. The results showed that at 0 h, the expression of ssc-miR-451, ssc-miR-486, and ssc-miR-1285 in the TK group was significantly increased compared with the other groups, and there was no significant difference between the JK and CK group, respectively. At 12 h, there was no significant difference in the expression of ssc-miR-451 and ssc-miR-1285 between the TK group and other groups, and the expression of ssc-miR-486 was significantly increased in the TK group. The above results indicated that, with the passage of perfusion time involving the pig kidney grafts, the expression of ssc-miR-451, ssc-miR-1285, and ssc-miR-486 gradually decreased, and there was no significant difference in the expression of ssc-miR-451 and ssc-miR-1285 at 12 h among the perfusion groups. The expression of ssc-miR-486 at 12 h in the TK group was significantly higher than in the other groups (Figure 2c). These results suggested that the three common miRNAs may play a role in the TK preservation method and were primarily active in the early stage.

Furthermore, the results of an intra-group comparison showed that at 0 and 12 h, there was nearly no change in the expression of ssc-miR-451, ssc-miR-1285, and ssc-miR-486 in the CK or JK groups, and expression was significantly down-regulated in the TK group (Figure 2d). This indicated that in the isolated kidney perfusion process, the differences in the expression of ssc-miR-451, ssc-miR-1285, and ssc-miR-486 could be paramount for distinguishing the TK from other perfusion methods at the molecular level; however, the specific role and mechanism require further investigation. The three perfusion methods could be replaced with one other, but miRNAs with a conservative function differed significantly between the TK, CK, and JK groups, suggesting that the CK method may be inferior to the TK or JK method.

### 3.3 The effects of miRNAs on the metabolism and proliferation of HK2 cells

**Figure 3.**
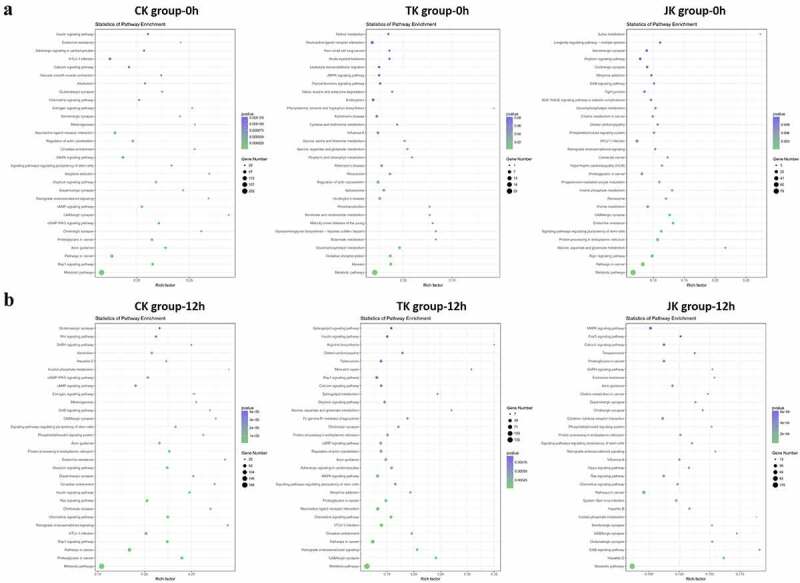
Bubble maps of the KEGG signaling pathway enrichment analysis of the perfusion groups at 0 (a) and 12 (b) h.

To explore the mechanisms of action of several differentially expressed miRNAs in kidney tissue using different perfusion methods, GO gene function enrichment analysis and KEGG signaling pathway enrichment analysis were performed. The GO gene function enrichment analysis results showed that there was essentially no difference in the cellular components, molecular function, or biological processes following perfusion among all the groups. The KEGG signaling pathway enrichment analysis results indicated that the most significant changes following perfusion had occurred in intracellular metabolism signaling pathways, such as the mitogen-activated protein kinase (MAPK), cyclic adenosine monophosphate (cAMP), Cyclic Guanosine monophosphate/protein kinase G (cGMP-PKG), and gamma-aminobutyric acid (GABBA) pathways in all groups at 0 and 12 h (Figure 3). The above results revealed that the cellular components, the molecular functions, and the biological processes of the isolated kidneys using different perfusion preservation methods were very similar and changes to cell metabolism were prominent.

**Figure 4.**
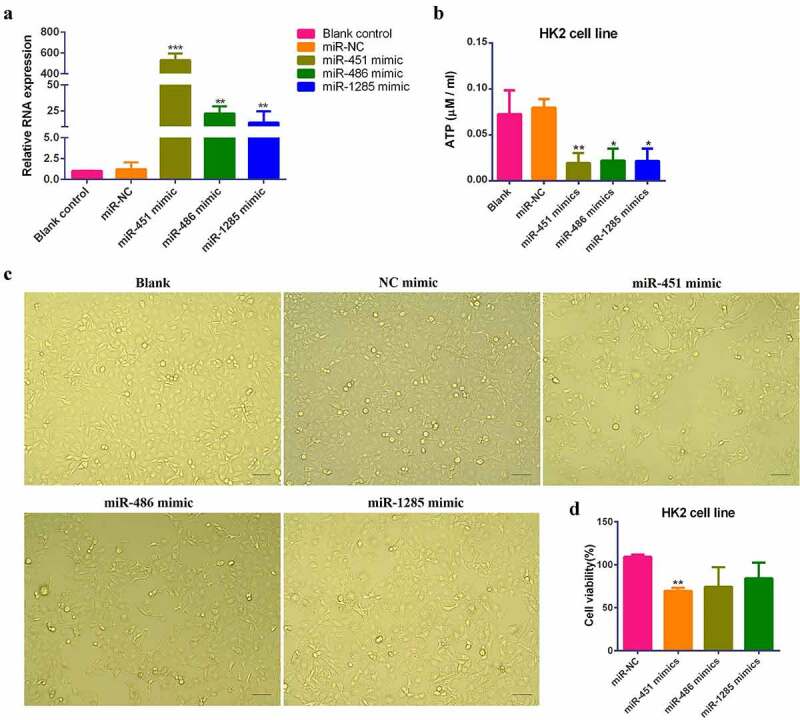
(a) The results of the qPCR assays after the transfection of the HK2 cells with three miRNA mimics. (b) The detection results of the ATP energy kits after transfection of the HK2 cells with miRNA mimics. (c) A morphological diagram of the HK2 cells after transfection with miRNA mimics; eyepiece x10 + objective x10. (d) The results of the MTT assays after transfection with miRNA mimics.

To verify the effects of the aforementioned miRNAs on the ATP metabolism of kidney cells, a miR-negative control, has-miR-451, has-miR-1285, and has-miR-486 mimic were transfected into renal tubular epithelial cells (HK2), respectively, and the ATP content in each group was detected using ATP energy metabolism kits. The qPCR results indicated significant miRNA overexpression (Figure 4a). The detection results of the kits showed that the ATP content in the HK2 cells significantly decreased after the overexpression of the miRNAs (Figure 4b). In addition, based on the proliferation of the HK2 cells after transfection and MTT assays, the proliferation of the HK2 cells was significantly inhibited following the overexpression of miR-451 (Figure 4c and d). The above results suggest that several miRNAs (miR-451, miR-1285, and miR-486), particularly miR-451, play an important role in the ATP energy metabolism of HK2 cells.

## 4. Discussion

Several studies have shown the potential of miRNAs as new biomarkers for transplant rejection reactions as well as their ability to regulate immune response following the transplantation of solid organs. In reports on renal IRI, Anglicheau et al. found that the levels of miR-142-5p, miR-155, and miR-223 in kidney grafts could help predict T-cell-mediated rejection [12]. Soltaninejad et al. found that the levels of miR-142-3p and miR-223 in peripheral blood mononuclear cells and the blood mononuclear cells of biopsy tissue could help identify acuteorgan rejection [13]. Lorenzen reported that the level of miR-10a in the urine of organ-rejected patients was significantly elevated, and the levels of miR-10b and miR-210 in the urine of organ-rejected patients were down-regulated compared with a control group [14]. Recently, a small study reported that the levels of miR-21 and miR-200b in urine could help distinguish kidney transplant patients with interstitial fibrosis and the renal tubular atrophy of grafts from kidney transplant patients with stable graft function [15]. The above-mentioned evidence demonstrates that miRNAs have great potential as new biomarkers for kidney transplant rejection.

Additionally, miR-451 plays a role in inflammation, oxidative stress, autophagy, and other physiological and pathological processes [16–18]. During organ IRI, miR-451 showed potential neuroprotective effects on this type of injury in stroke patients and mice [19]. Furthermore, miR-451 was reported to be associated with immune rejection in organ transplants [20]. Conversely, miR-486 was involved in the regulation of the nuclear factor-kappa B signal pathway, inflammatory response, and p53-mediated mitochondrial apoptosis signaling [21,22]. In addition, as a biomarker for the early detection of the chronic antibody-mediated rejection of kidney transplants, miR-486 mediated rejection responses in kidney transplantation cases [23] while miR-1285 regulated the transcription and co-transcription factors of multiple cell-signaling pathways, including the yes-associated protein, the JUN gene, and p53 protein. Its current studies are focused on cancer [24–26], and additional functions have not been reported. In the current study, the expression of ssc-miR-451, ssc-miR-1285, and ssc-miR-486 was significantly down-regulated over time in the TK group, and the differences in the other groups were less significant than those in this group. Three functional small molecules, i.e., MiR-451, miR-1285, and miR-486, reflected extremely conservative functions. The authors speculate that they may also inhibit the inflammatory response to protect renal hypoxic-ischemic injury in pig kidney tissue.

Studies showed that cells treated with GC7 (a targeted drug for activating amino-acid synthase) can reversely induce a shift from cell metabolism to glycolysis and reshape mitochondria, resulting in the down-regulation of the expression and activity of the respiratory chain complex and mitochondrial silencing [27]. Additionally, GC7 treatment can also reduce the reactive oxygen generated by cells under oxygen deficiency and decrease the mitochondrial oxygen consumption of cells in mice. In addition, the intraperitoneal injection of rats with GC7 significantly lowered the level of epinephrine eIF5A and protected kidneys from IRI [10]. The intravenous injection of GC7 into animal models prior to organ transplantation improved postoperative graft function recovery and late graft functioning and reduced renal interstitial fibrosis after transplantation [27,28]. These studies indicate that metabolic obstruction plays an important role in the hypoxic-ischemic injury of transplanted kidneys and is one of the major causes of postoperative graft dysfunction. The results of the present study showed that most differentially expressed miRNAs were associated with cell metabolism, which was consistent with an existing report stating the function recovery of transplanted kidneys could be improved through metabolism intervention. The above results suggest that different perfusion methods can be replaced with one another, and the functional activity of transplanted kidneys can be increased by metabolism intervention before transplantation.

In summary, the results of this study showed that there were few differences between the kidney tissue sampled from different perfusion groups. At the molecular level, three small molecules with conservative functions (miR-451, miR-1285, and miR-486) were differentially expressed in different perfusion groups, and their expression in the TK group was significantly better than in the other groups. Most differentially expressed miRNAs were associated with cell metabolism, including MAPK, cAMP, cGMP-PKG, GABA, and other intracellular metabolism signaling pathways. Furthermore, miRNAs, including miR-451, miR-1285, and miR-486, played important roles in the ATP energy metabolism of HK2 cells, thus regulating cell survival.

## 5. Conclusion

The results of this study suggest that the essentially equivalent changes in early tissue samples and at molecular levels, as well as similar changes in cell functioning and signaling pathways, may serve as an internal mechanism for the mutual replacement of three different perfusion methods, and that differences in the functional activity of three kidney transplant types can be changed by metabolism intervention prior to transplantation.
